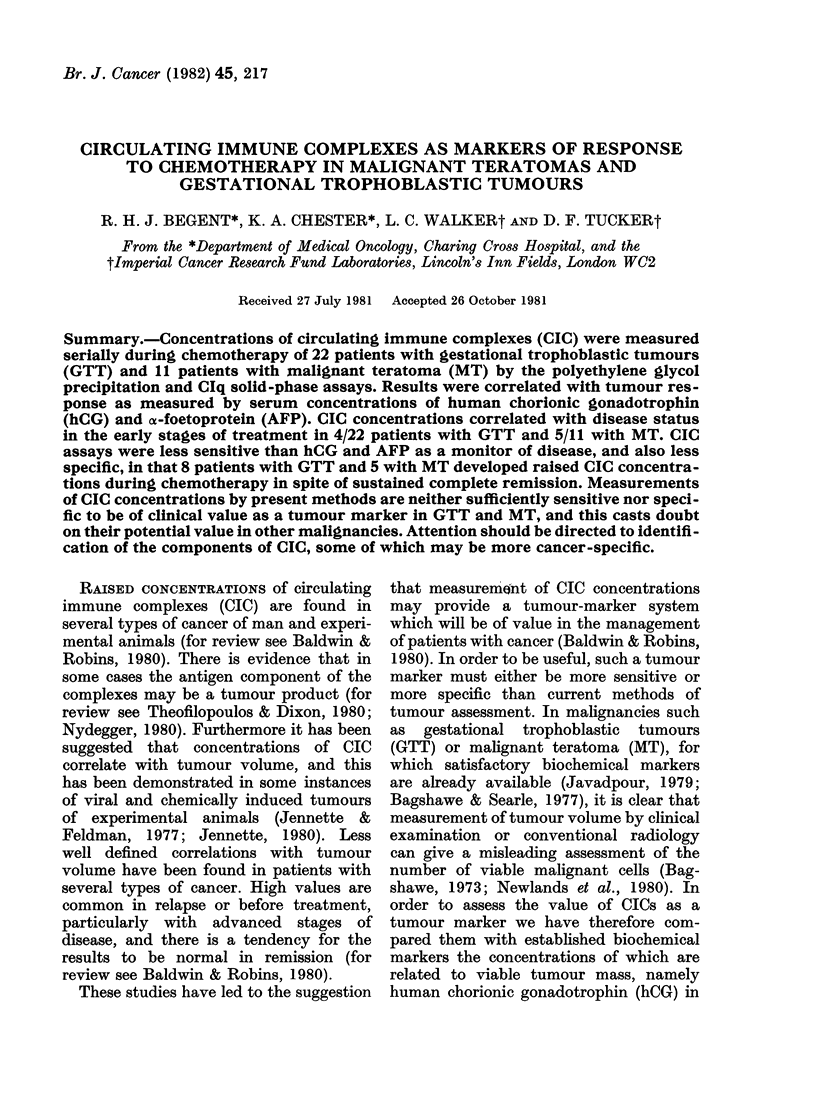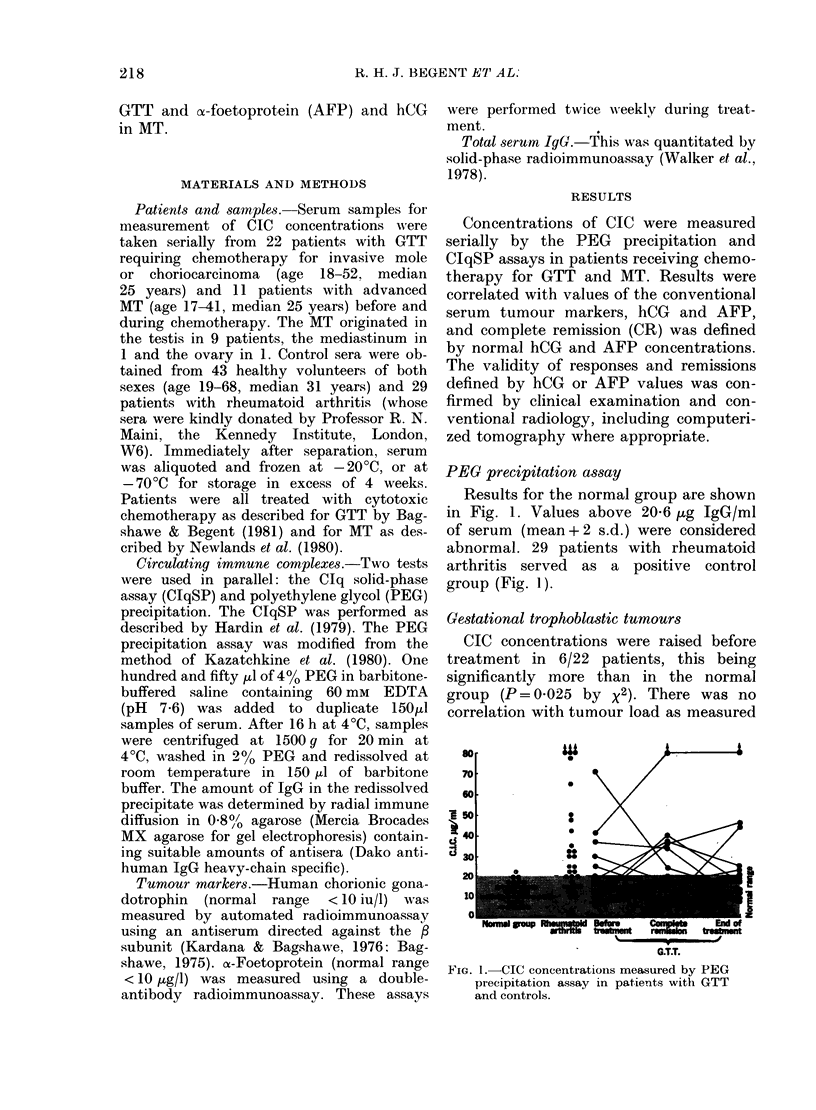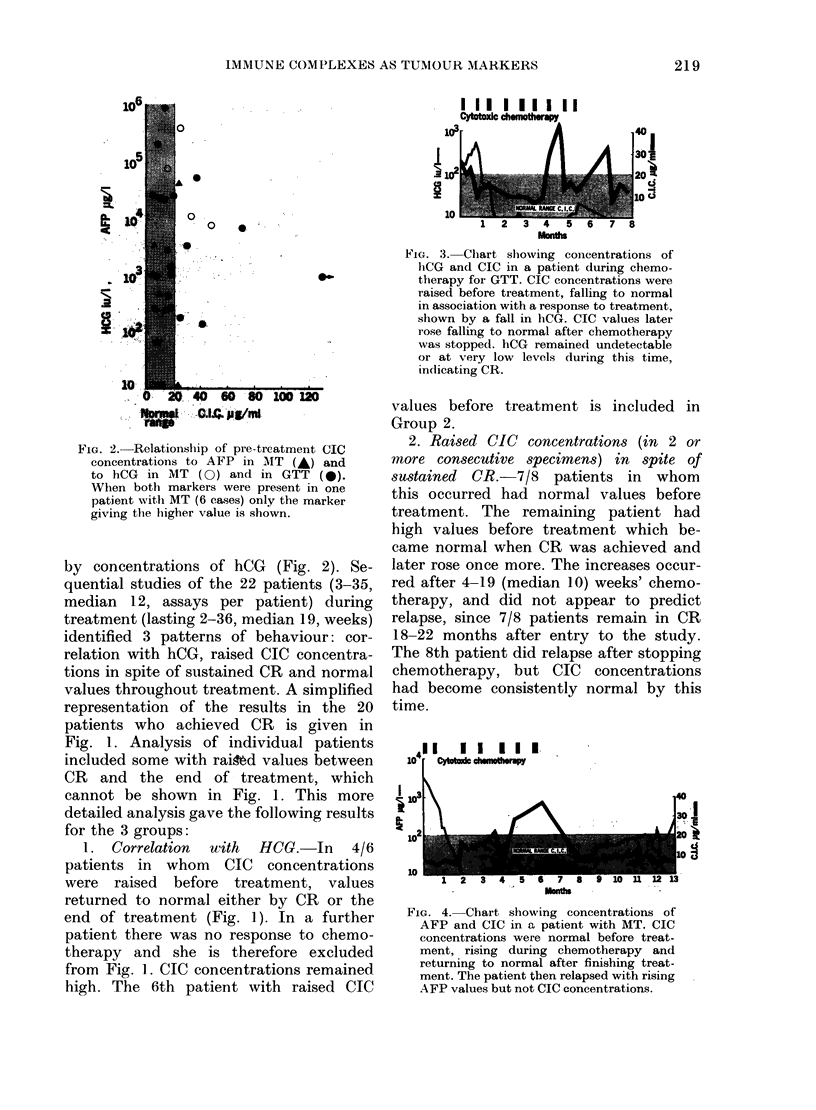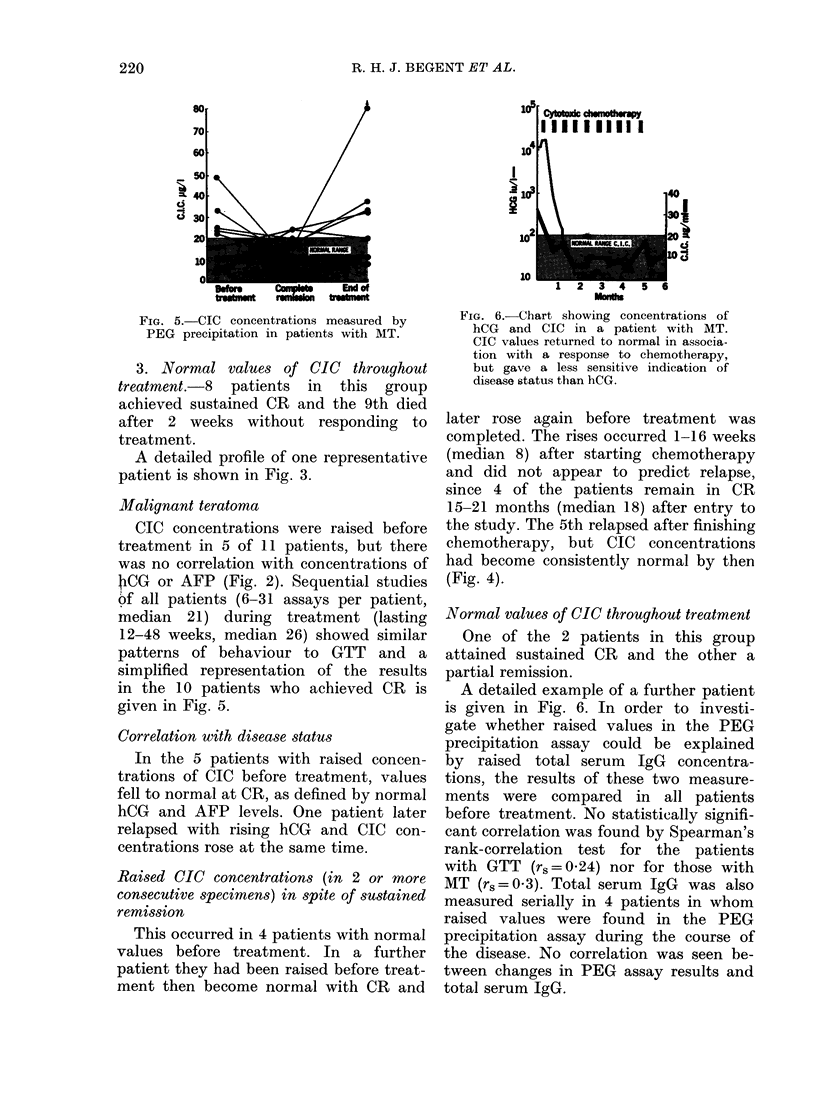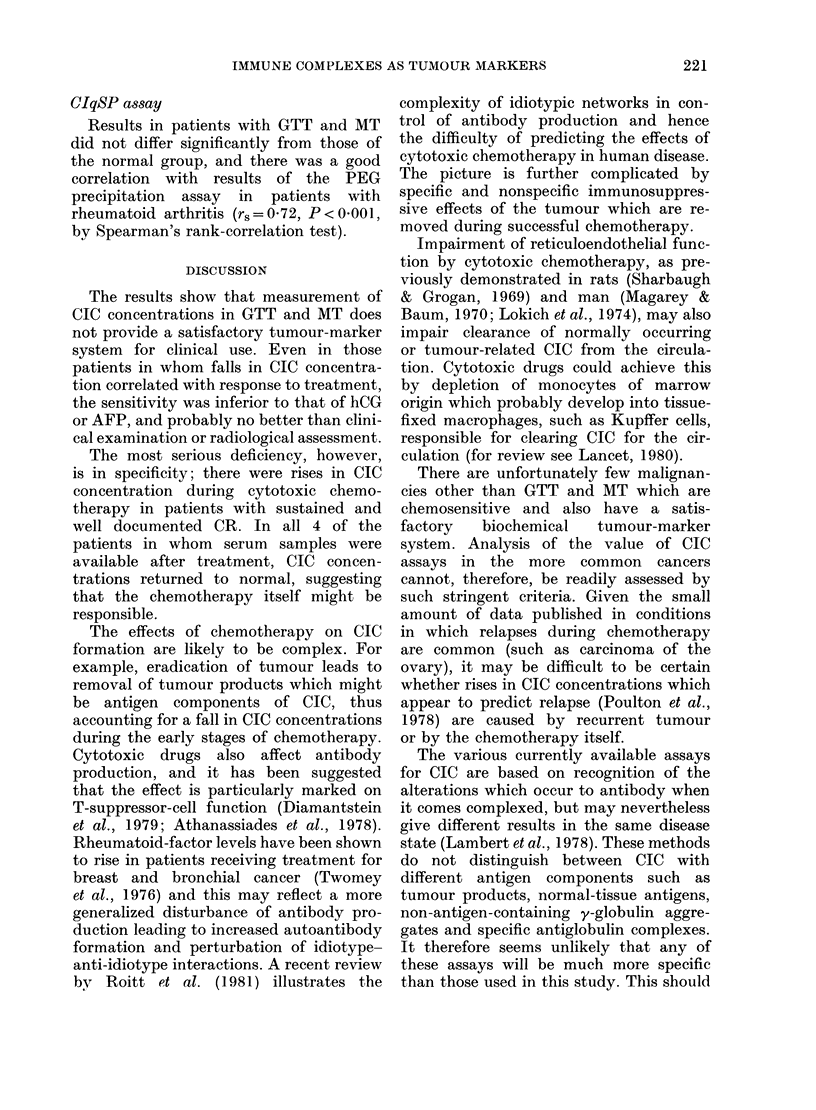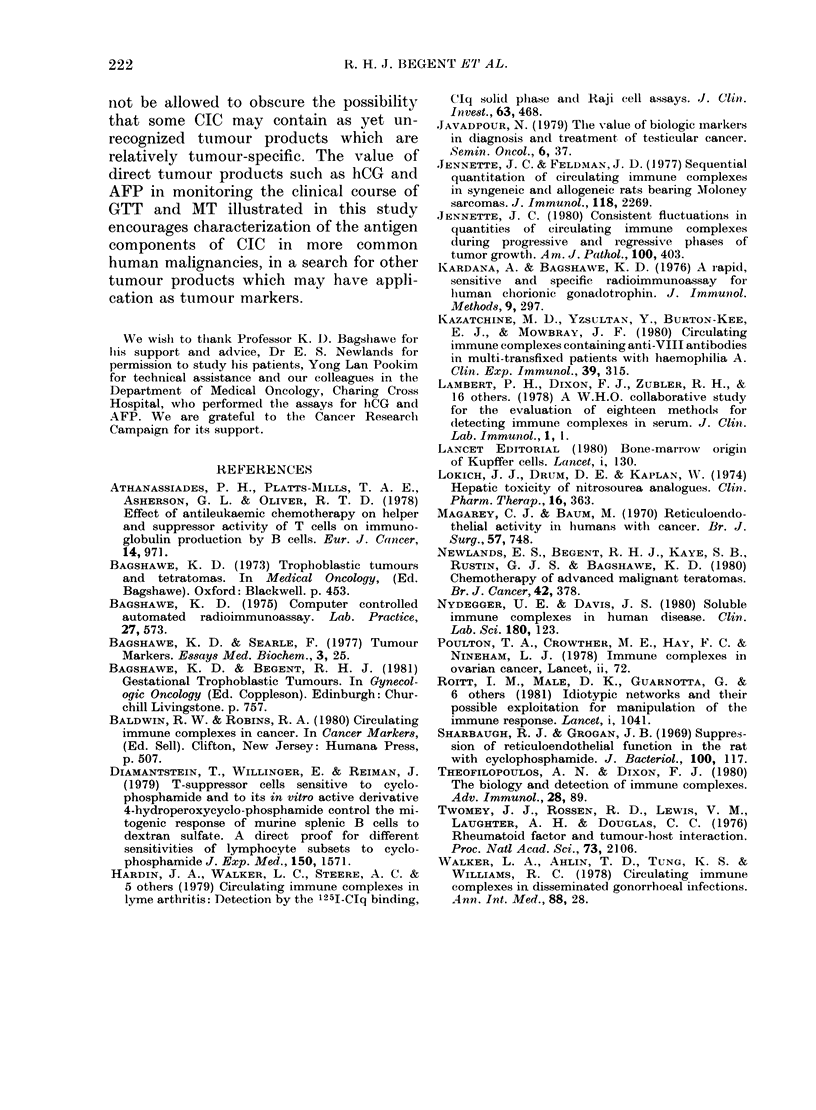# Circulating immune complexes as markers of response to chemotherapy in malignant teratomas and gestational trophoblastic tumours.

**DOI:** 10.1038/bjc.1982.36

**Published:** 1982-02

**Authors:** R. H. Begent, K. A. Chester, L. C. Walker, D. F. Tucker

## Abstract

Concentrations of circulating immune complexes (CIC) were measured serially during chemotherapy of 22 patients with gestational trophoblastic tumours (GTT) and 11 patients with malignant teratoma (MT) by the polyethylene glycol precipitation and CIq solid-phase assays. Results were correlated with tumour response as measured by serum concentrations of human chorionic gonadotrophin (hCG) and alpha-foetoprotein (AFP). CIC concentrations correlated with disease status in the early stages of treatment in 4/22 patients with GTT and 5/11 with MT. CIC assays were less sensitive than hCG and AFP as a monitor of disease, and also less specific, in that 8 patients with GTT and 5 with MT developed raised CIC concentrations during chemotherapy in spite of sustained complete remission. Measurements of CIC concentrations by present methods are neither sufficiently sensitive nor specific to be of clinical value as a tumour marker in GTT and MT, and this casts doubt on their potential value in other malignancies. Attention should be directed to identification of the components of CIC, some of which may be more cancer-specific.


*Br. J. Cancer* (1982) **45**, 217

### CIRCULATING IMMUNE COMPLEXES AS MARKERS OF RESPONSE TO CHEMOTHERAPY IN MALIGNANT TERATOMAS AND GESTATIONAL TROPHOBLASTIC TUMOURS

R. H. J. BEGENT*, K. A. CHESTER*, L. C. WALKER† AND D. F. TUCKER†

*From the *Department of Medical Oncology, Charing Cross Hospital, and the †Imperial Cancer Research Fund Laboratories, Lincoln's Inn Fields, London WC2*

Received 27 July 1981 Accepted 26 October 1981

**Summary.—Concentrations of circulating immune complexes (CIC) were measured serially during chemotherapy of 22 patients with gestational trophoblastic tumours (GTT) and 11 patients with malignant teratoma (MT) by the polyethylene glycol precipitation and CIq solid-phase assays. Results were correlated with tumour response as measured by serum concentrations of human chorionic gonadotrophin (hCG) and α-foetoprotein (AFP). CIC concentrations correlated with disease status in the early stages of treatment in 4/22 patients with GTT and 5/11 with MT. CIC assays were less sensitive than hCG and AFP as a monitor of disease, and also less specific, in that 8 patients with GTT and 5 with MT developed raised CIC concentrations during chemotherapy in spite of sustained complete remission. Measurements of CIC concentrations by present methods are neither sufficiently sensitive nor specific to be of clinical value as a tumour marker in GTT and MT, and this casts doubt on their potential value in other malignancies. Attention should be directed to identification of the components of CIC, some of which may be more cancer-specific.**

RAISED CONCENTRATIONS of circulating immune complexes (CIC) are found in several types of cancer of man and experimental animals (for review see Baldwin & Robins, 1980). There is evidence that in some cases the antigen component of the complexes may be a tumour product (for review see Theofilopoulos & Dixon, 1980; Nydegger, 1980). Furthermore it has been suggested that concentrations of CIC correlate with tumour volume, and this has been demonstrated in some instances of viral and chemically induced tumours of experimental animals (Jennette & Feldman, 1977; Jennette, 1980). Less well defined correlations with tumour volume have been found in patients with several types of cancer. High values are common in relapse or before treatment, particularly with advanced stages of disease, and there is a tendency for the results to be normal in remission (for review see Baldwin & Robins, 1980).

These studies have led to the suggestion that measurement of CIC concentrations may provide a tumour-marker system which will be of value in the management of patients with cancer (Baldwin & Robins, 1980). In order to be useful, such a tumour marker must either be more sensitive or more specific than current methods of tumour assessment. In malignancies such as gestational trophoblastic tumours (GTT) or malignant teratoma (MT), for which satisfactory biochemical markers are already available (Javadpour, 1979; Bagshawe & Searle, 1977), it is clear that measurement of tumour volume by clinical examination or conventional radiology can give a misleading assessment of the number of viable malignant cells (Bagshawe, 1973; Newlands *et al.*, 1980). In order to assess the value of CICs as a tumour marker we have therefore compared them with established biochemical markers the concentrations of which are related to viable tumour mass, namely human chorionic gonadotrophin (hCG) in

GTT and α-foetoprotein (AFP) and hCG in MT.

### MATERIALS AND METHODS

*Patients and samples.*—Serum samples for measurement of CIC concentrations were taken serially from 22 patients with GTT requiring chemotherapy for invasive mole or choriocarcinoma (age 18–52, median 25 years) and 11 patients with advanced MT (age 17–41, median 25 years) before and during chemotherapy. The MT originated in the testis in 9 patients, the mediastinum in 1 and the ovary in 1. Control sera were obtained from 43 healthy volunteers of both sexes (age 19–68, median 31 years) and 29 patients with rheumatoid arthritis (whose sera were kindly donated by Professor R. N. Maini, the Kennedy Institute, London, W6). Immediately after separation, serum was aliquoted and frozen at −20°C, or at −70°C for storage in excess of 4 weeks. Patients were all treated with cytotoxic chemotherapy as described for GTT by Bagshawe & Begent (1981) and for MT as described by Newlands *et al.* (1980).

*Circulating immune complexes.*—Two tests were used in parallel: the CIq solid-phase assay (CIqSP) and polyethylene glycol (PEG) precipitation. The CIqSP was performed as described by Hardin *et al.* (1979). The PEG precipitation assay was modified from the method of Kazatchkine *et al.* (1980). One hundred and fifty μl of 4% PEG in barbitone-buffered saline containing 60 mM EDTA (pH 7·6) was added to duplicate 150μl samples of serum. After 16 h at 4°C, samples were centrifuged at 1500 *g* for 20 min at 4°C, washed in 2% PEG and redissolved at room temperature in 150 μl of barbitone buffer. The amount of IgG in the redissolved precipitate was determined by radial immune diffusion in 0·8% agarose (Mercia Brocades MX agarose for gel electrophoresis) containing suitable amounts of antisera (Dako anti-human IgG heavy-chain specific).

*Tumour markers.*—Human chorionic gonadotrophin (normal range < 10 iu/l) was measured by automated radioimmunoassay using an antiserum directed against the β subunit (Kardana & Bagshawe, 1976; Bagshawe, 1975). α-Foetoprotein (normal range < 10 μg/l) was measured using a double-antibody radioimmunoassay. These assays were performed twice weekly during treatment.

*Total serum IgG.*—This was quantitated by solid-phase radioimmunoassay (Walker *et al.*, 1978).

### RESULTS

Concentrations of CIC were measured serially by the PEG precipitation and CIqSP assays in patients receiving chemotherapy for GTT and MT. Results were correlated with values of the conventional serum tumour markers, hCG and AFP, and complete remission (CR) was defined by normal hCG and AFP concentrations. The validity of responses and remissions defined by hCG or AFP values was confirmed by clinical examination and conventional radiology, including computerized tomography where appropriate.

### *PEG precipitation assay*

Results for the normal group are shown in Fig. 1. Values above 20·6 μg IgG/ml of serum (mean + 2 s.d.) were considered abnormal. 29 patients with rheumatoid arthritis served as a positive control group (Fig. 1).

### *Gestational trophoblastic tumours*

CIC concentrations were raised before treatment in 6/22 patients, this being significantly more than in the normal group ($P = 0{\cdot}025$ by $\chi^2$). There was no correlation with tumour load as measured

FIG. 1.—CIC concentrations measured by PEG precipitation assay in patients with GTT and controls.

FIG. 2.—Relationship of pre-treatment CIC concentrations to AFP in MT (▲) and to hCG in MT (○) and in GTT (●). When both markers were present in one patient with MT (6 cases) only the marker giving the higher value is shown.

FIG. 3.—Chart showing concentrations of hCG and CIC in a patient during chemotherapy for GTT. CIC concentrations were raised before treatment, falling to normal in association with a response to treatment, shown by a fall in hCG. CIC values later rose falling to normal after chemotherapy was stopped. hCG remained undetectable or at very low levels during this time, indicating CR.

by concentrations of hCG (Fig. 2). Sequential studies of the 22 patients (3–35, median 12, assays per patient) during treatment (lasting 2–36, median 19, weeks) identified 3 patterns of behaviour: correlation with hCG, raised CIC concentrations in spite of sustained CR and normal values throughout treatment. A simplified representation of the results in the 20 patients who achieved CR is given in Fig. 1. Analysis of individual patients included some with raised values between CR and the end of treatment, which cannot be shown in Fig. 1. This more detailed analysis gave the following results for the 3 groups:

1. *Correlation with HCG.*—In 4/6 patients in whom CIC concentrations were raised before treatment, values returned to normal either by CR or the end of treatment (Fig. 1). In a further patient there was no response to chemotherapy and she is therefore excluded from Fig. 1. CIC concentrations remained high. The 6th patient with raised CIC values before treatment is included in Group 2.

2. *Raised CIC concentrations (in 2 or more consecutive specimens) in spite of sustained CR.*—7/8 patients in whom this occurred had normal values before treatment. The remaining patient had high values before treatment which became normal when CR was achieved and later rose once more. The increases occurred after 4–19 (median 10) weeks' chemotherapy, and did not appear to predict relapse, since 7/8 patients remain in CR 18–22 months after entry to the study. The 8th patient did relapse after stopping chemotherapy, but CIC concentrations had become consistently normal by this time.

FIG. 4.—Chart showing concentrations of AFP and CIC in a patient with MT. CIC concentrations were normal before treatment, rising during chemotherapy and returning to normal after finishing treatment. The patient then relapsed with rising AFP values but not CIC concentrations.

FIG. 5.—CIC concentrations measured by PEG precipitation in patients with MT.

FIG. 6.—Chart showing concentrations of hCG and CIC in a patient with MT. CIC values returned to normal in association with a response to chemotherapy, but gave a less sensitive indication of disease status than hCG.

3. *Normal values of CIC throughout treatment.*—8 patients in this group achieved sustained CR and the 9th died after 2 weeks without responding to treatment.

A detailed profile of one representative patient is shown in Fig. 3.

*Malignant teratoma*

CIC concentrations were raised before treatment in 5 of 11 patients, but there was no correlation with concentrations of hCG or AFP (Fig. 2). Sequential studies of all patients (6–31 assays per patient, median 21) during treatment (lasting 12–48 weeks, median 26) showed similar patterns of behaviour to GTT and a simplified representation of the results in the 10 patients who achieved CR is given in Fig. 5.

*Correlation with disease status*

In the 5 patients with raised concentrations of CIC before treatment, values fell to normal at CR, as defined by normal hCG and AFP levels. One patient later relapsed with rising hCG and CIC concentrations rose at the same time.

*Raised CIC concentrations (in 2 or more consecutive specimens) in spite of sustained remission*

This occurred in 4 patients with normal values before treatment. In a further patient they had been raised before treatment then become normal with CR and later rose again before treatment was completed. The rises occurred 1–16 weeks (median 8) after starting chemotherapy and did not appear to predict relapse, since 4 of the patients remain in CR 15–21 months (median 18) after entry to the study. The 5th relapsed after finishing chemotherapy, but CIC concentrations had become consistently normal by then (Fig. 4).

*Normal values of CIC throughout treatment*

One of the 2 patients in this group attained sustained CR and the other a partial remission.

A detailed example of a further patient is given in Fig. 6. In order to investigate whether raised values in the PEG precipitation assay could be explained by raised total serum IgG concentrations, the results of these two measurements were compared in all patients before treatment. No statistically significant correlation was found by Spearman's rank-correlation test for the patients with GTT ($r_S = 0{\cdot}24$) nor for those with MT ($r_S = 0{\cdot}3$). Total serum IgG was also measured serially in 4 patients in whom raised values were found in the PEG precipitation assay during the course of the disease. No correlation was seen between changes in PEG assay results and total serum IgG.

*ClqSP assay*

Results in patients with GTT and MT did not differ significantly from those of the normal group, and there was a good correlation with results of the PEG precipitation assay in patients with rheumatoid arthritis ($r_S = 0{\cdot}72$, $P < 0{\cdot}001$, by Spearman's rank-correlation test).

### DISCUSSION

The results show that measurement of CIC concentrations in GTT and MT does not provide a satisfactory tumour-marker system for clinical use. Even in those patients in whom falls in CIC concentration correlated with response to treatment, the sensitivity was inferior to that of hCG or AFP, and probably no better than clinical examination or radiological assessment.

The most serious deficiency, however, is in specificity; there were rises in CIC concentration during cytotoxic chemotherapy in patients with sustained and well documented CR. In all 4 of the patients in whom serum samples were available after treatment, CIC concentrations returned to normal, suggesting that the chemotherapy itself might be responsible.

The effects of chemotherapy on CIC formation are likely to be complex. For example, eradication of tumour leads to removal of tumour products which might be antigen components of CIC, thus accounting for a fall in CIC concentrations during the early stages of chemotherapy. Cytotoxic drugs also affect antibody production, and it has been suggested that the effect is particularly marked on T-suppressor-cell function (Diamantstein *et al.*, 1979; Athanassiades *et al.*, 1978). Rheumatoid-factor levels have been shown to rise in patients receiving treatment for breast and bronchial cancer (Twomey *et al.*, 1976) and this may reflect a more generalized disturbance of antibody production leading to increased autoantibody formation and perturbation of idiotype–anti-idiotype interactions. A recent review by Roitt *et al.* (1981) illustrates the complexity of idiotypic networks in control of antibody production and hence the difficulty of predicting the effects of cytotoxic chemotherapy in human disease. The picture is further complicated by specific and nonspecific immunosuppressive effects of the tumour which are removed during successful chemotherapy.

Impairment of reticuloendothelial function by cytotoxic chemotherapy, as previously demonstrated in rats (Sharbaugh & Grogan, 1969) and man (Magarey & Baum, 1970; Lokich *et al.*, 1974), may also impair clearance of normally occurring or tumour-related CIC from the circulation. Cytotoxic drugs could achieve this by depletion of monocytes of marrow origin which probably develop into tissue-fixed macrophages, such as Kupffer cells, responsible for clearing CIC for the circulation (for review see Lancet, 1980).

There are unfortunately few malignancies other than GTT and MT which are chemosensitive and also have a satisfactory biochemical tumour-marker system. Analysis of the value of CIC assays in the more common cancers cannot, therefore, be readily assessed by such stringent criteria. Given the small amount of data published in conditions in which relapses during chemotherapy are common (such as carcinoma of the ovary), it may be difficult to be certain whether rises in CIC concentrations which appear to predict relapse (Poulton *et al.*, 1978) are caused by recurrent tumour or by the chemotherapy itself.

The various currently available assays for CIC are based on recognition of the alterations which occur to antibody when it comes complexed, but may nevertheless give different results in the same disease state (Lambert *et al.*, 1978). These methods do not distinguish between CIC with different antigen components such as tumour products, normal-tissue antigens, non-antigen-containing $\gamma$-globulin aggregates and specific antiglobulin complexes. It therefore seems unlikely that any of these assays will be much more specific than those used in this study. This should not be allowed to obscure the possibility that some CIC may contain as yet unrecognized tumour products which are relatively tumour-specific. The value of direct tumour products such as hCG and AFP in monitoring the clinical course of GTT and MT illustrated in this study encourages characterization of the antigen components of CIC in more common human malignancies, in a search for other tumour products which may have application as tumour markers.

We wish to thank Professor K. D. Bagshawe for his support and advice, Dr E. S. Newlands for permission to study his patients, Yong Lan Pookim for technical assistance and our colleagues in the Department of Medical Oncology, Charing Cross Hospital, who performed the assays for hCG and AFP. We are grateful to the Cancer Research Campaign for its support.

### REFERENCES

ATHANASSIADES, P. H., PLATTS-MILLS, T. A. E., ASHERSON, G. L. & OLIVER, R. T. D. (1978) Effect of antileukaemic chemotherapy on helper and suppressor activity of T cells on immunoglobulin production by B cells. *Eur. J. Cancer*, **14**, 971.

BAGSHAWE, K. D. (1973) Trophoblastic tumours and tetratomas. In *Medical Oncology*, (Ed. Bagshawe). Oxford: Blackwell. p. 453.

BAGSHAWE, K. D. (1975) Computer controlled automated radioimmunoassay. *Lab. Practice*, **27**, 573.

BAGSHAWE, K. D. & SEARLE, F. (1977) Tumour Markers. *Essays Med. Biochem.*, **3**, 25.

BAGSHAWE, K. D. & BEGENT, R. H. J. (1981) Gestational Trophoblastic Tumours. In *Gynecologic Oncology* (Ed. Coppleson). Edinburgh: Churchill Livingstone. p. 757.

BALDWIN, R. W. & ROBINS, R. A. (1980) Circulating immune complexes in cancer. In *Cancer Markers*, (Ed. Sell). Clifton, New Jersey: Humana Press, p. 507.

DIAMANTSTEIN, T., WILLINGER, E. & REIMAN, J. (1979) T-suppressor cells sensitive to cyclophosphamide and to its *in vitro* active derivative 4-hydroperoxycyclo-phosphamide control the mitogenic response of murine splenic B cells to dextran sulfate. A direct proof for different sensitivities of lymphocyte subsets to cyclophosphamide *J. Exp. Med.*, **150**, 1571.

HARDIN, J. A., WALKER, L. C., STEERE, A. C. & 5 others (1979) Circulating immune complexes in lyme arthritis: Detection by the $^{125}$I-CIq binding, CIq solid phase and Raji cell assays. *J. Clin. Invest.*, **63**, 468.

JAVADPOUR, N. (1979) The value of biologic markers in diagnosis and treatment of testicular cancer. *Semin. Oncol.*, **6**, 37.

JENNETTE, J. C. & FELDMAN, J. D. (1977) Sequential quantitation of circulating immune complexes in syngeneic and allogeneic rats bearing Moloney sarcomas. *J. Immunol.*, **118**, 2269.

JENNETTE, J. C. (1980) Consistent fluctuations in quantities of circulating immune complexes during progressive and regressive phases of tumor growth. *Am. J. Pathol.*, **100**, 403.

KARDANA, A. & BAGSHAWE, K. D. (1976) A rapid, sensitive and specific radioimmunoassay for human chorionic gonadotrophin. *J. Immunol. Methods*, **9**, 297.

KAZATCHINE, M. D., YZSULTAN, Y., BURTON-KEE, E. J., & MOWBRAY, J. F. (1980) Circulating immune complexes containing anti-VIII antibodies in multi-transfixed patients with haemophilia A. *Clin. Exp. Immunol.*, **39**, 315.

LAMBERT, P. H., DIXON, F. J., ZUBLER, R. H., & 16 others. (1978) A W.H.O. collaborative study for the evaluation of eighteen methods for detecting immune complexes in serum. *J. Clin. Lab. Immunol.*, **1**, 1.

LANCET EDITORIAL (1980) Bone-marrow origin of Kupffer cells. *Lancet*, i, 130.

LOKICH, J. J., DRUM, D. E. & KAPLAN, W. (1974) Hepatic toxicity of nitrosourea analogues. *Clin. Pharm. Therap.*, **16**, 363.

MAGAREY, C. J. & BAUM, M. (1970) Reticuloendothelial activity in humans with cancer. *Br. J. Surg.*, **57**, 748.

NEWLANDS, E. S., BEGENT, R. H. J., KAYE, S. B., RUSTIN, G. J. S. & BAGSHAWE, K. D. (1980) Chemotherapy of advanced malignant teratomas. *Br. J. Cancer*, **42**, 378.

NYDEGGER, U. E. & DAVIS, J. S. (1980) Soluble immune complexes in human disease. *Clin. Lab. Sci.* **180**, 123.

POULTON, T. A., CROWTHER, M. E., HAY, F. C. & NINEHAM, L. J. (1978) Immune complexes in ovarian cancer, Lancet, ii, 72.

ROITT, I. M., MALE, D. K., GUARNOTTA, G. & 6 others (1981) Idiotypic networks and their possible exploitation for manipulation of the immune response. *Lancet*, i, 1041.

SHARBAUGH, R. J. & GROGAN, J. B. (1969) Suppression of reticuloendothelial function in the rat with cyclophosphamide. *J. Bacteriol.*, **100**, 117.

THEOFILOPOULOS, A. N. & DIXON, F. J. (1980) The biology and detection of immune complexes. *Adv. Immunol.*, **28**, 89.

TWOMEY, J. J., ROSSEN, R. D., LEWIS, V. M., LAUGHTER, A. H. & DOUGLAS, C. C. (1976) Rheumatoid factor and tumour-host interaction. *Proc. Natl Acad. Sci.*, **73**, 2106.

WALKER, L. A., AHLIN, T. D., TUNG, K. S. & WILLIAMS, R. C. (1978) Circulating immune complexes in disseminated gonorrhoeal infections. *Ann. Int. Med.*, **88**, 28.